# Structural vs. functional mechanisms of duplicate gene loss following whole genome doubling

**DOI:** 10.1186/1471-2105-16-S17-S9

**Published:** 2015-12-07

**Authors:** David Sankoff, Chunfang Zheng, Baoyong Wang, Carlos Fernando Buen Abad Najar

**Affiliations:** 1 Department of Mathematics and Statistics, University of Ottawa, Ottawa, Canada; 2 Facultad de Ciencias, Universidad Nacional Autónoma de México, Avenida Universidad 3000, Distrito Federal, México

**Keywords:** whole genome doubling, fractionation, run length statistics, gene loss, gene duplication

## Abstract

**Background:**

The loss of duplicate genes - fractionation - after whole genome doubling (WGD) is the subject to a debate as to whether it proceeds gene by gene or through deletion of multi-gene chromosomal segments.

**Results:**

WGD produces two copies of every chromosome, namely two identical copies of a sequence of genes. We assume deletion events excise a geometrically distributed number of consecutive genes with mean *µ *≥ 1, and these events can combine to produce single-copy runs of length *l*. If *µ *= 1, the process is gene-by-gene. If *µ *> 1, the process at least occasionally excises more than one gene at a time. In the latter case if deletions overlap, the later one simply extends the existing run of single-copy genes. We explore aspects of the predicted distribution of the lengths of single-copy regions analytically, but resort to simulations to show how observing run lengths *l *allows us to discriminate between the two hypotheses.

**Conclusions:**

Deletion run length distributions can discriminate between gene-by-gene fractionation and deletion of segments of geometrically distributed length, even if *µ *is only slightly larger than 1, as long as the genome is large enough and fractionation has not proceeded too far towards completion.

## Background

The process of whole genome doubling (WGD) gives rise to two copies of each chromosome in a genome, containing the same genes in the same order. Through an attrition mechanism known as fractionation, one of each pair of duplicate genes is lost over evolutionary time. The rare deletion of both copies of a gene can be excluded from our considerations of the interleaving patterns of deletions from duplicated regions first discovered by Wolfe and Shields [1]. The retention of one copy of each pair is what differentiates the WGD/fractionation model from approaches to gene duplication, insertion and deletion in the study of comparative genomics, pioneered by El-Mabrouk [2].

An important biological controversy arises from the question of whether duplicated genes are deleted through random excision - elimination of excess DNA - namely the deletion of chromosomal segments containing one or more genes [3], which we term the "structural" mechanism, or through gene-by gene events such as epigenetic silencing and pseudogenization [4], which are "functional" mechanisms. This question is important to evolutionary theory because it speaks directly to the role of WGD, and gene duplication in general, in disrupting gene order, in creating functional innovation, and in the radiation of new species. It is a question of whether selection operates on the level of simply permitting non-lethal deletions or whether more subtle effects are in play, such as dosage balance of interacting genes.

This debate may be formulated in terms of deletion events removing a number *X* of contiguous genes, where *X *is drawn from a geometric distribution γ with mean *µ*. Here the one-at-a-time deletion model is represented by *µ *= 1, while the random number of deletions at a time holds if *µ *> 1. In the latter case, the possibility of two overlapping events is handled by a biologically realistic additive run-length assumption.

In this paper, we investigate the discrimination problem of choosing between the two models based on deletion run-length statistics (resulting from overlapping deletion events). This involves comparing an observed genome containing single-copy genes, originally members of duplicate pairs, to the predictions of the models for *µ *= 1 and for *µ *> 1. This requires knowledge of the run-length distribution, given a total number of deleted genes and remaining duplicate pairs. While this is easily calculated for the case *µ *= 1, the the distribution for the opposing scenario *µ *> 1 is not known.

In the first part of this paper, we analyze aspects of the deletion run-length distribution *ψ* when *µ *> 1 for the deletion-length distribution γ, including some new and surprising analytical results, the clearest of which pertain to a continuous analog of the problem. We then show why it is difficult to describe *ψ* in closed form or other easily computable format. In the second part, we simulate the distribution and carry out a study of the discrimination problem for various values of *µ*, genome size *N* and *θ*, the proportion of undeleted genes at time t. We conclude with a discussion of the remaining mathematical problems to be solved before the method can be applied to data from real WGD descendants.

## Results

### The models

For modeling purposes, we consider a doubled genome made up, at the outset, of a pair of identical linear chromosomes each containing genes *g*_1_, . . . , *g*_*N *_, where *N* is large enough so that we can neglect end effects - particular behaviors near *g_1 _and g_N _*. At each time *t* = 1, 2, . . ., one such doubled gene g_i _is chosen at random, and a value *X* is chosen from a geometric distribution *γ *with mean *µ*. If *X = a*, then g_i_, *g*_*i*+1_, . . . , *g*_*i*+*a−1 *_are deleted from one of the genomes - they become single-copy genes - unless some of these are already single-copy. In the latter case, we skip existing single-copy genes and proceed to convert the next double-copy genes we encounter until a total of a double-copy genes have been converted to single-copy. Note that this overlapping of deletion events never occurs if *µ *= 1 since, in this case, by definition, exactly one double-copy gene is selected and deleted in each step. For simplicity, we assume all deletions take place from one and the same genome. In a more complete model, deletion events would occur on one or the other chromosome, with probabilities *φ *and 1 − *φ *[5].

The "skipping" procedure, introduced in [6], is a natural way to model the deletion process, since deletion of part of a chromosome and the subsequent rejoining of the chromosome directly before and directly after the deleted fragment means that this fragment is no longer "visible" to the deletion process. As observers, however, we have a record of the deleted genes, as one copy of each gene must be retained in the genome.

Overlapping deletion events and skipping result in the creation of runs of single-copy genes whose length is the sum of a number of geometric variables. The sum of *r* identical geometric variables produces a negative binomial distribution with parameter *r*, but the skipping process does not involve the sum of identical random variables, since a deletion with a large value of *a* is more likely to overlap an existing single copy region than a deletion with small *a*. Thus, at any point of time *t *> 0, the distribution ψ_t _of single-copy run lengths will tend to contain a higher frequency of runs of length 1, and of very long runs, than would be generated by the negative binomial. On the other hand, the distribution of run lengths of the remaining double-copy genes is geometrically distributed with a probability distribution ρ_t_, where the mean *ν_t _*decreases with t [5,6].

### Analysis of overlap probabilities

An attempt to determine *ψ*_*t *_analytically starts with the calculation of how many deletion events have overlapped to form a run of single-copy genes at time *t*. In [6], we derived a formula to predict whether a deletion event would create a new run of single-copy genes, probability *p*_0_; overlap exactly one existing run, thus extending it without changing the total number of runs, probability *p*_1_; overlap two runs, producing one larger combined run in place of the two pre-existing ones, probability *p*_2_; and so on. Other probabilities deal with the events that a run "touches" a pre-existing run without overlapping it. These probabilities all depend solely on *γ *and *ρ_t_*. For example, we examine the case of *p_0_*. The other probabilities are all formulated in analogous ways.

The proportion of genes in single-copy runs of length *l *is $l\rho_{\text{t}}\left(l\right)/\nu_{\text{t}}$, where $\nu_{t}=\sum_{{}_{l>0}}l\rho_{t}\left(l\right)$. The probability *p*_0 _that a deletion event falls within a run of double-copy genes without deleting the terms at either end is

(1) $$\begin{array}{cc} p_{0} & =\underset{l>2}{\sum }\frac{l\rho_{t}\left(l\right)}{v_{t}}\overset{l-1}{\underset{j=2}{\sum }}\frac{1}{l}\overset{l-j}{\underset{a=1}{\sum }}\gamma \left(a\right)\\ & =\frac{1}{v_{t}}\underset{l>2}{\sum }\rho_{t}\left(l\right)\overset{l-1}{\underset{j=2}{\sum }}\overset{l-j}{\underset{a=1}{\sum }}\gamma \left(a\right)\\ & =\frac{1}{v_{t}}\underset{l>2}{\sum }\rho_{t}\left(l\right)\overset{l-2}{\underset{a=1}{\sum }}\left(l-a-1\right)\gamma \left(a\right) \end{array}$$

where *j* indexes the starting position of the deletion within a run of length *l*, and a is the number of genes deleted in the event.

This formula requires quadratic computing time, but the *p_i _*for higher *i*, require polynomial time of degree *i *+ 2. Here we exemplify with *p*_0 _to show that these probabilities can in fact be reduced to closed form, so that computing time is a negligible constant. The formula in (1), when expanded, consists of a number of partial sums of the geometric distributions *γ *and *ρ*_*t *_and means of these distributions, all of which are readily reduced to closed form, plus sums of terms of the form ${\left[\left(\text{1 }-\frac{1}{\mu }\right)\left(\text{1 }-\frac{1}{v_{t}}\right)\right]}^{l}$ and $l{\left[\left(\text{1 }-\frac{1}{\mu }\right)\left(\text{1 }-\frac{1}{v_{t}}\right)\right]}^{l}$, which themselves can be considered in terms of a geometric distribution with mean ζ, where

(2) $$\text{1}-\frac{1}{\zeta }=\left(\text{1}-\frac{1}{\mu }\right)\left(\text{1}-\frac{1}{v_{t}}\right)$$

Then (1) reduces to:

(3) $$p_{0}=\frac{{\left(\nu_{t}-1\right)}^{2}}{\left(\mu +\;\nu_{t}-1\right)\nu_{t}}\;$$

For large ν*_t_*, i.e., during the early stage of the process,

(4) $$p_{0}\approx \frac{v_{t}}{\mu +v_{t}}\left(1-\frac{1}{v_{t}}\right)$$

Typically, *µ *is somewhere between 1 and 2, [3,4], and *ν_t _*of the order of 10^3 ^or 10^4^. Thus *p*_0 _is initially only slightly less than 1 but declines rapidly as *ν_t _*decreases exponentially.

We proceed in an analogous way to derive closed forms for *p_1_, p_2_*, . . ., but it is perhaps more instructive here to present the continuous version of the deletion process. Here the two identical chromosomes at time *t *= 0 are linear segments, long enough in comparison with the other parameters of the model so that end effects can be ignored. At each time *t = *1, 2, . . . , a random point *g* is chosen on the chromosome, and a value *X* is chosen from an exponential distribution

(5) $$f\left(a\right)=\frac{1}{\mu }e^{-\frac{a}{\mu }}\;,a\geq 0$$

with mean *µ*. If *X* = *a*, then the segment [*g, g +a*] is deleted from one of the genomes - [*g, g + a*] becomes a single-copy region - unless part of it is already single-copy. In the latter case, we skip existing single-copy regions and proceed to convert the next double-copy region we encounter until a total measure a of double-copy regions have been converted to single-copy.

In analogy with the discrete model, the combined length of the remaining double-copy segments is exponentially distributed according to probability distribution *σ_t_*, with a mean *ν_t _*that decreases with *t*.

The proportion of undeleted regions accounted for by segments of length *ldl *is $\frac{l\sigma \left(l\right)}{v_{t}}dl$, where $\nu_{t}=\overset{\infty }{\underset{0}{\int }}l\sigma \left(l\right)dl$. Then the probability p_0 _that a deletion event falls completely within an undeleted segment is

(6) $$p_{0}=\int_{l=0}^{\infty }\frac{l\sigma_{t}\left(l\right)}{v_{t}}\int_{x=0}^{l}\frac{1}{l}\int_{y=0}^{l-x}f\left(y\right)dy\;dx\;dl$$

Carrying out the integrations, we find

(7) $$p_{0}=\frac{v_{t}}{\mu +v_{t}}$$

which is reminiscent of the relation (4) in the discrete case with large ν*_t_*.

The probability *p*_1_ that a deletion event overlaps exactly one existing run of deletions is:

(8) $$p_{1}=\frac{1}{v_{t}}\int_{l=0}^{\infty }\int_{z=0}^{\infty }\sigma_{t}\left(l\right)\sigma_{t}\left(z\right)\int_{x=0}^{l}\int_{y=l-x}^{l-x+z}f\left(y\right)dydxdzdl$$

(9) $$=\frac{v_{t}}{\mu +v_{t}}\;\frac{\mu }{\mu +v_{t}}$$

It can be proved by induction that the probability a deletion event overlaps exactly *q* existing runs of deletions is:

(10) $$p_{q}=\frac{v_{t}}{\mu +v_{t}}{\left(\frac{\mu }{\mu +v_{t}}\right)}^{q}$$

Thus we have the surprisingly uncomplicated result that the number q of pre-existing runs of single-copy regions overlapped by a new deletion event is geometrically distributed on q = 0, 1, . . . with parameter *µ*/(*µ *+ ν*_t_*).

### On the run-length distribution

**Figure 1:**
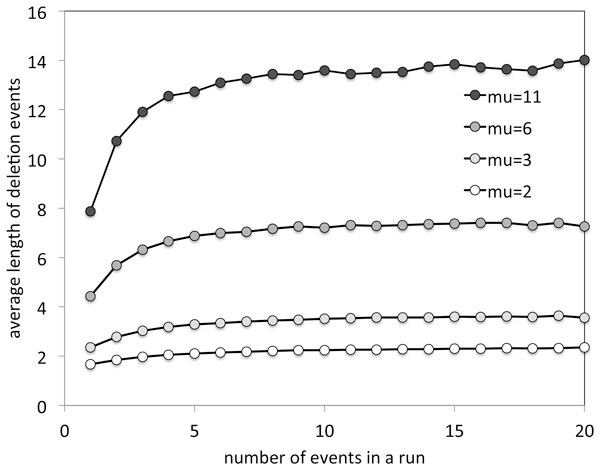
**Non-independence of overlapping events**. Simulation of the number of overlapping deletion events making up a single-copy region, when 70% of the genes are single copy. With a large number of events in a run, the individual events tend to have greater lengths. From [6].

Although having a closed form for *p*_*q *_constitutes progress towards the computation of the run-length distribution *ψ*_*t*_, or eventually towards some analytical results on it, how to find this distribution remains a difficult question. As mentioned in the previous section, long deletion events will be involved in more skipping than small ones. This is illustrated in Figure 1, where runs built from a small number of events tend to be composed of shorter deletions, especially when *µ *is large. Had we just added independent samples from a geometric distribution, the curves in the figure would have been horizontal lines.

How to account for the distorting effect of skipping on the run-length distribution will require additional insight and research. In the interim, we may use simulations to study the discrimination problem.

### Simulations

We first simulated the fractionation process for all combinations of the following parameter values:

• gene number *N *= 100 to 900, in steps of 100.

• *µ *= 1.0 to 2.4, in steps of 0.1.

• Proportion of the genes deleted, 1 − *θ *= 0.1 to 0.9, in steps of 0.1.

For each combination of the parameters *µ*, N and *θ*, we calculated the distribution of run lengths *l *for single-copy regions, and similarly for double-copy regions. The simulation was repeated 1000 times and the frequencies of length (*l *= 1,2,3,...) of runs of deleted genes were averaged over the 1000 trials to get a reasonably accurate estimate of the cumulative *F*_*µ,N,1−θ *_. Similarly we estimated the cumulative G_*µ,N,1−θ *_for runs of remaining double-copy genes.

Once the cumulative distributions were established, we then carried out the actual discrimination study. For each value of *µ *and *N*, we sampled 1000 new individual trajectories of the deletion process until 1 − *θ *= 90 % of the genes were deleted. For each value of 1 − *θ *= 0.1, 0.2, . . . , 0.9, we created "bins" corresponding to the fifteen values of *µ *for which we had constructed cumulatives. Then for each sample S*_i_*, at each 1 − *θ *= 0.1, . . . , 0.9 we counted the frequency of runs of deleted genes of length = 1, 2, . . . and constructed a cumulative distribution. We calculated the Kolmogorov-Smirnov statistic $D_{\mu ,N,1-\theta }^{\left(Si\right)}$between the sample cumulative and the previously established distribution *F*_*µ,N,1−θ *_for each fifteen values of *µ *and assigned the sample to the bin corresponding to the minimal value of this statistic, which was our estimate $\hat{\mu }$ for that sample.

**Figure 2:**
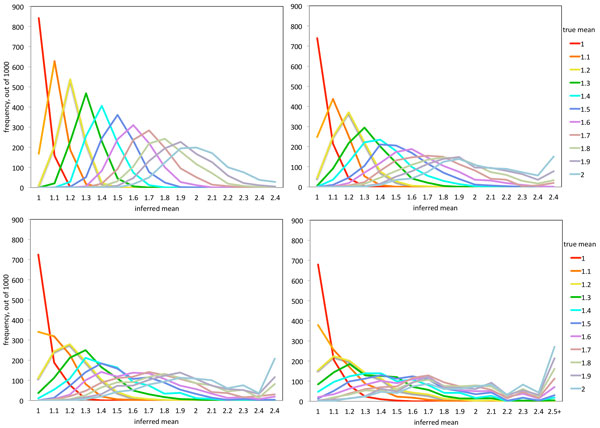
**Discrimination between models based on run lengths of deleted genes**. Frequency of $\hat{\mu }$, the value for which $D_{\mu ,N,1-\theta }^{{}^{\left(S_{i}\;\right)}}$between the sample cumulative and the distribution *F*_*µ,N,1−θ *_is minimal. All data involve a proportion of 1 − *θ *= 0.20 deleted genes. Top left: *N *= 900. Top right: *N *= 300. Bottom left: *N *= 200. Bottom right: *N *= 100.

Figure 2 shows the distributions of $\hat{\mu }$ for the 1000 samples *S*_1_, . . . , *S*_1000_. The four panels are the results of *N *= 900, 300, 200 and 100. A separate distribution is drawn for each of the trial values of *µ *used to generate the samples. For *N *= 900 (top left), there is a clear pattern of the mode of the distribution to occur at the same value of *µ *that generated the data, though the distributions become more spread out for higher values of *µ*. The same pattern may be seen for *N *= 300 (top right), though considerably degraded. This loss of accuracy of $\hat{\mu }$ continues through *N *= 200 (bottom left) and *N *= 100 (bottom right), where the modes for $\hat{\mu }$ when *µ *= 1.1 are in the *µ *= 1.0 bin.

**Figure 3:**
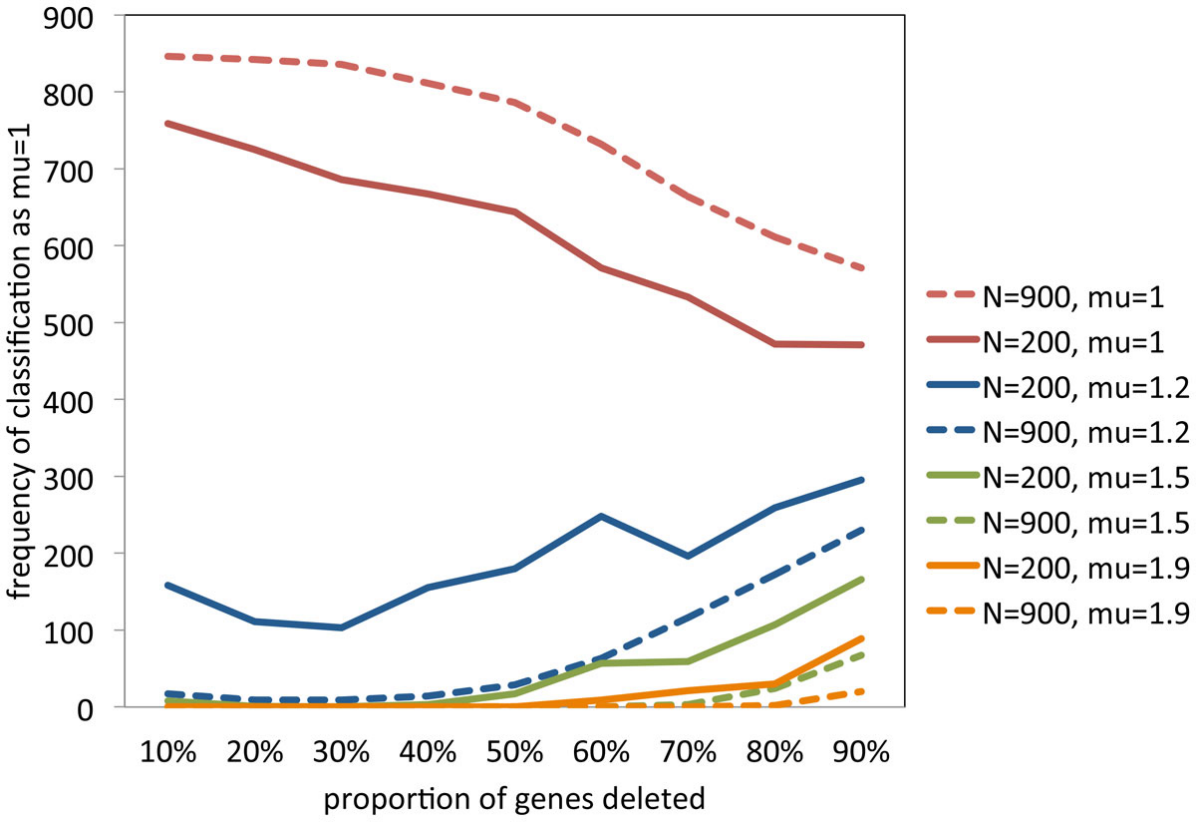
**Assessment of tests**. Frequency of $\hat{\mu }=1$ as a function of 1 − *θ*, for *µ *= 1 (functional hypothesis) and various *µ *> 1 (structural hypothesis), for *N *= 900 and 200.

With all four values of *N* in Figure 2, the most accurate inference is made for *µ *= 1, the gene-by-gene model. This brings us back to the original problem of discriminating between the gene-by-gene "functional model" (*µ *= 1) and the random excision "structural" model (*µ *> 1). Figure 3 shows the frequency with which we estimate $\hat{\mu }=\text{1}$, for various values of *µ *and *N *= 200 or 900, as a function of 1 − *θ*, the proportion of genes deleted. The upper curves in the figure show that we can correctly identify the *µ *= 1 model around 70-85% of the time; more for *N *= 900 and less for *N *= 200, as long as 1 − *θ *< 50%. In other words, the type I error of a test of H_0 _: *µ *= 1 against *H*_1 _: *µ *> 1 with these parameters and procedures, is about 15-30%. The lower curves show that incorrectly inferring $\hat{\mu }=\text{1}$ occurs around 20% of the time when *µ *= 1.2, but very rarely for *µ *= 1.9 or even *µ *= 1.5, until 1 − *θ *begins to exceed 50%. In other words, if now *H_m _*: *µ *= *m*, for some constant *m *> 1, is the null hypothesis and *H*_0 _is the alternative, then the type I error is very small unless *m* is very close to 1 (e.g., *m *= 1.2) or 1 − *θ *is large (e.g., >50% if *m *= 1.5).

**Figure 4:**
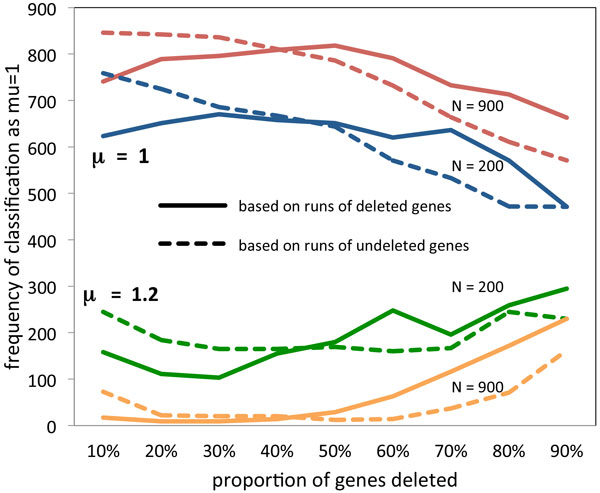
**Comparison of double-copy and single-copy analyses**. Frequency of $\hat{\mu }=1$ as a function of 1 − *θ*, for *µ *= 1 and 1.2 and *N *= 900 and 200. Results based on runs of single-copy (deleted) genes contrasted with results from double-copy (undeleted) genes.

Up to now, we have examined only runs of single-copy genes. What of the runs of remaining double-copy genes? Figure 4 compares some of the results from the same simulations as Figure 3, but using the cumulative *G_µ,N,1−θ _*for runs of double-copy genes as well as *F*_*µ,N,1−θ *_for runs of single-copy genes. The main observation is that the double-copy approach systematically infers *µ *= 1 with higher frequency for small values of 1 − *θ*, whether or not this inference corresponds to the generating *µ*. It systematically infers *µ *= 1 with lower frequency for large values of 1 − *θ*, again whether or not this is correct. These simulations establish ranges of values of *N, µ *and 1 − *θ *for which we can and cannot discriminate between the two models.

## Conclusions

In this work we have made some progress in deriving the run-length distribution *ψ_t _*for single-copy regions, although this problem is still not completely resolved. From an analytical point of view, it is unexpected and interesting that in the continuous version of the problem, the number of pre-existing runs overlapped by a deletion event follows a geometric distribution.

The simulation study showed the much greater difficulty in distinguishing between the structural and functional models when the mean *µ *of the deletion size distribution is 1.1 rather than 1.9, when N is 100 rather than 900, and when the proportion of genes deleted is bigger than 50% rather than less than 40%. The latter effect is also apparent in empirical studies [7].

Our simulation results are based on a "binning" strategy for determining $\hat{\mu }$ for the purposes of discrimination, rather than an asymmetrical testing approach comparing the hypotheses *µ *= 1 and *µ *> 1. This is justified by the lack of any biological significance, and high rates of error, in comparing μ=1+∈ and *µ *= 1 for very small  ∈, as well as the global picture it offers of the degradation of discriminatory power as a function of *µ, N *and *θ*.

This work has for the first time enabled the systematic discrimination between the two models of duplicate deletion following WGD. Future research will continue on the analytical determination of *ψ*_*t *_as well as extension to the "two-sided" deletion models proposed in [5]. Eventually, we will have to allow processes of genome rearrangement to disrupt runs of single-copy genes or double-copy genes, as in [7]. It is these kinds of model that will eventually be useful for analyzing data from real genomes.

## Competing interests

The authors declare that they have no competing interests.

## Authors' contributions

All authors participated in the research, wrote the paper, read and approved the manuscript.

## Declarations

Publication of this article was funded by NSERC Discovery grant DG 8867-2008 RGPIN.

This article has been published as part of *BMC Bioinformatics *Volume 16 Supplement 17, 2015: Selected articles from the Fourth IEEE International Conference on Computational Advances in Bio and medical Sciences (ICCABS 2014): Bioinformatics. The full contents of the supplement are available online at http://www.biomedcentral.com/bmcbioinformatics/supplements/16/S17.
